# A novel approach to facilitate guidewire placement in endoscopic ultrasound-guided biliary drainage

**DOI:** 10.1055/a-2689-3740

**Published:** 2025-09-09

**Authors:** Cancan Zhou, Zheng Wang, Zheng Wu, Hao Sun, Jie Hao

**Affiliations:** 1 162798 Hepatobiliary Surgery, The First Affiliated Hospital of Xi'an Jiaotong University, Xi’an, China

In cases that are refractory to endoscopic retrograde cholangiopancreatography (ERCP), endoscopic ultrasound-guided biliary drainage (EUS-BD) has emerged as a preferred alternative 1 . For a hepaticogastrostomy (HGS) or hepaticogastric anastomosis with stent placement, puncture of the left intrahepatic bile duct, typically segment II (B2) or segment III (B3), via the stomach is standard; however, a common challenge, irrespective of the segment chosen, is inadvertent advancement of the guidewire into the peripheral intrahepatic ducts. instead of toward the common hepatic duct (CHD) 2 3 . While partial needle retraction into the hepatic parenchyma may facilitate guidewire redirection in some cases, this maneuver is often suboptimal 4 . To address this challenge, we employed a rotatable needle knife to redirect the guidewire ( Video 1 ).

Endoscopic ultrasound-guided biliary drainage (EUS-BD) is successfully performed after initial attempts to advance the guidewire into the common hepatic duct (CHD) through a 19-gauge needle placed into the B3 intrahepatic bile duct failed, and a rotatable needle knife was instead used, which allowed the guidewire to be advanced into the distal common bile duct and, ultimately, into the CHD, with EUS-BD completed by insertion of a stent.Video 1

A 56-year-old man presented with obstructive jaundice secondary to pancreatic head malignancy. Following unsuccessful ERCP, he underwent EUS-BD via the stomach. Under EUS guidance, the B3 intrahepatic bile duct was punctured using a 19-gauge needle ( Fig. 1 **a** ) and a 0.035-inch guidewire was introduced. The guidewire was initially advanced peripherally within the intrahepatic ducts ( Fig. 1 **b** ). Despite multiple attempts to redirect it toward the CHD by partially withdrawing the needle, the maneuver was unsuccessful ( Fig. 2 **a** ).

**Fig. 1:**
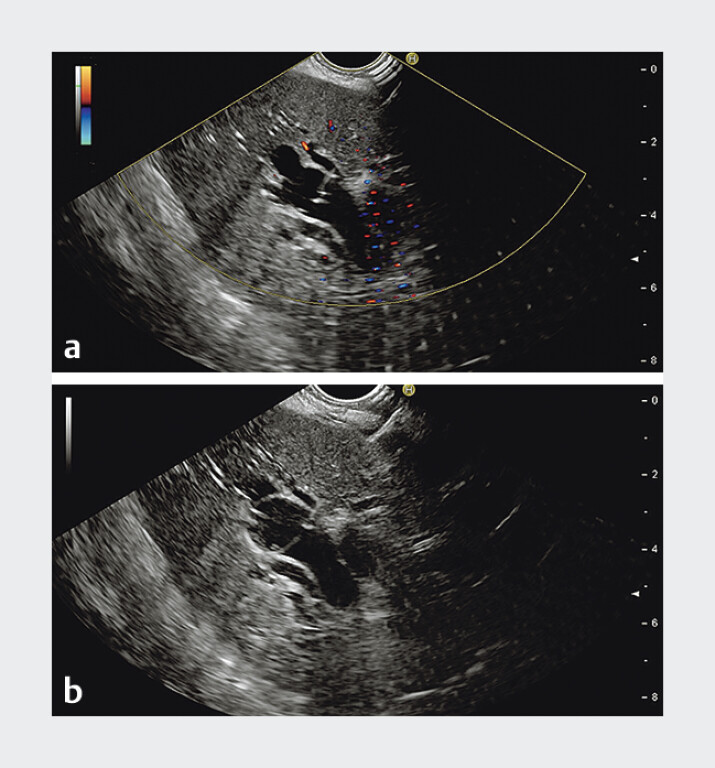
Endoscopic ultrasound images showing: **a** the intrahepatic bile duct being punctured by a 19G needle; **b** the guidewire being advanced peripherally within the intrahepatic ducts.

**Fig. 2:**
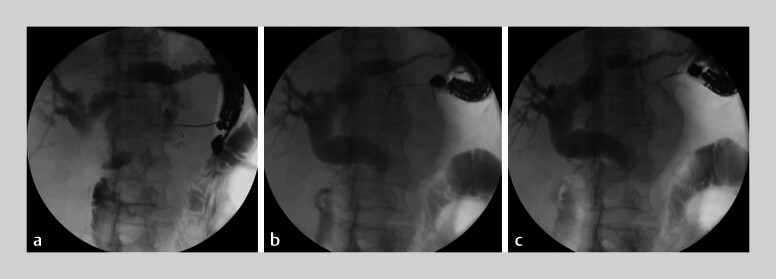
Fluoroscopic images showing: **a** unsuccessful guidewire placement; **b** the rotatable needle knife being used; **c** successful placement of the guidewire after rotation of the needle knife within the duct.

Subsequently, we replaced the needle with a rotatable needle knife, which was advanced into the bile duct ( Fig. 2 **b** ). Under combined fluoroscopic and EUS guidance, the needle knife was rotated 180° within the duct, which allowed the guidewire to be successfully redirected toward the hilum ( Fig. 2 **c** ). The guidewire was then advanced into the distal common bile duct, enabling completion of the hepaticogastric anastomosis with stent placement. The patient recovered uneventfully with no procedure-related adverse events.

This case demonstrates that the rotatable needle knife can serve as a safe and effective adjunctive technique for guidewire redirection during EUS-BD when conventional wire manipulation fails.

Endoscopy_UCTN_Code_TTT_1AS_2AH

## Notes

Endoscopy E-Videos https://eref.thieme.de/e-videos
*E-Videos* is an open access online section of the journal *Endoscopy* , reporting on interesting cases and new techniques in gastroenterological endoscopy. All papers include a high-quality video and are published with a Creative Commons CC-BY license. Endoscopy E-Videos qualify for HINARI discounts and waivers and eligibility is automatically checked during the submission process. We grant 100% waivers to articles whose corresponding authors are based in Group A countries and 50% waivers to those who are based in Group B countries as classified by Research4Life (see: https://www.research4life.org/access/eligibility/ ). This section has its own submission website at https://mc.manuscriptcentral.com/e-videos .

## References

[1] Hatamaru K Kitano M EUS-guided biliary drainage for difficult cannulation Endosc Ultrasound 2019 8 01 S67 S71 10.4103/eus.eus_60_19 31897382 PMC6896436

[2] Kadkhodayan K Irani S EUS-guided hepaticogastrostomy: practical tips and tricks VideoGIE 2024 9 417 424 10.1016/j.vgie.2024.05.015 39429912 PMC11489514

[3] Ogura T Higuchi K Technical review of developments in endoscopic ultrasound-guided hepaticogastrostomy Clin Endosc 2021 54 651 659 10.5946/ce.2021.020-KDDW 33896154 PMC8505184

[4] Ogura T Masuda D Takeuchi T Liver impaction technique to prevent shearing of the guidewire during endoscopic ultrasound-guided hepaticogastrostomy Endoscopy 2015 47 01 E583 E584 26649471 10.1055/s-0034-1393381

